# Customized registry tool for tracking adherence to clinical guidelines for head and neck cancers: protocol for a pilot study

**DOI:** 10.1186/s40814-020-0552-0

**Published:** 2020-02-07

**Authors:** Matthew D. Hickey, Sarah Lisker, Shauna Brodie, Eric Vittinghoff, Marika D. Russell, Urmimala Sarkar

**Affiliations:** 1 Department of Medicine, University of California San Francisco, School of Medicine, San Francisco, CA 94143 USA; 2 Center for Vulnerable Populations, University of California San Francisco, San Francisco, 94110 CA USA; 3 Department of Otolaryngology – Head and Neck Surgery, University of California San Francisco, School of Medicine, San Francisco, CA 94143 USA; 4 Department of Epidemiology and Biostatistics, University of California San Francisco, School of Medicine, San Francisco, CA 94143 USA

**Keywords:** Patient monitoring, Patient safety, Ambulatory care, Organizational interventions, Systems engineering

## Abstract

**Background:**

Despite recommendations for monitoring patients with chronic and high-risk conditions, gaps still remain. These gaps are exacerbated in outpatient care, where patients and clinicians face challenges related to care coordination, multiple electronic health records, and extensive follow-up. In addition, low-income and racial/ethnic minority populations that are disproportionately cared for in safety net settings are particularly at risk to lapses in monitoring.

**Methods:**

We aim to implement and evaluate a health information technology platform developed using systems engineering methodologies. The implementation is situated in a clinic that monitors patients with head and neck cancer within a large, urban, publicly funded hospital. Our study will evaluate the time it takes for patients to progress through key treatment milestones prior to and after implementation of the tool. We will use models controlling for secular trend to estimate the effect of the tool on improving timely and successful completion of guideline-based care processes.

**Discussion:**

This protocol details the evaluation of the effectiveness of a human-centered health information technology intervention on improving timely delivery of care for high-risk populations. Other settings, including those that face challenges related to limited resources to devote to safety programs and fragmented health information technology, may benefit from this approach.

**Trial registration:**

ClinicalTrials.gov, NCT03546322. “Customized Registry Tool for Tracking Adherence to Clinical Guidelines for Head and Neck Cancers.” Registered 1 June 2018.

## Background

Since the publication of “To Err is Human” and other subsequent reports by the Institute of Medicine, there has been increasing focus on harms caused by medical care [1–3]. Errors of omission, or failing to deliver needed treatment, are especially common in the ambulatory setting, where patients receive only 55% of recommended preventive care. Those with chronic conditions fare no better, receiving only 56% of recommended care [4].

Delivery of appropriate care in the ambulatory setting frequently requires monitoring and treatment over multiple visits and often extended periods of time, particularly for those with chronic diseases or complex conditions. Missed monitoring may put patients at high-risk of adverse effects of treatment or failed detection of progression of disease. In addition to periodic monitoring, patients with complex conditions may require coordination between multiple specialties and complex treatment regimens that require timely completion of needed steps. For example, patients with cancer often require extensive diagnostic workup, evaluation by an interdisciplinary group of providers, and coordination of multiple treatment modalities. In head and neck cancer, the National Comprehensive Cancer Network (NCCN) has published guidelines for recommended pre-treatment workup, treatment, and post-treatment monitoring [5]. Despite the presence of these guidelines, fewer than half of patients undergoing surgery for head and neck squamous cell carcinoma receive post-surgery radiation within the timeframe recommended by NCCN guidelines [6]. Unsurprisingly, studies show an association between delays in treatment initiation and increased mortality [7]. Treatment interruptions have also been associated with both persistent disease [8] and mortality [9]. Perhaps more concerning, the proportion of patients receiving guideline-based care has decreased over time, and patients with lower socioeconomic status who receive care regardless of insurance status or their ability to pay in safety net settings appear to be most vulnerable [6, 10]. Though reasons for failure to adhere to guidelines are complex, lapses are frequently the result of the inability of clinics and health systems to proactively identify patients who meet criteria for guideline-based diagnostic or therapeutic interventions [11].

Traditionally, face-to-face visits have been the primary means of monitoring patients and ensuring completion of treatment steps. However, given the frequency of errors of omission—missed cancer diagnoses constitute the leading cause of paid medical malpractice claims among outpatients—solutions to systematically identify upcoming and overdue monitoring across a population of patients are needed [12, 13]. Similar to cancer screening, which is now often based on electronic registries and outreach to patients rather than purely visit-based activity, there is an opportunity to use technology and team-based workflows to enhance outpatient monitoring for high-risk conditions.

As part of the process for developing the information technology (IT) tool used in this study, we conducted a series of qualitative interviews with practitioners in a number of clinics involved in delivering care to patients with chronic or complex medical conditions at high-risk of experiencing monitoring related medical errors. We applied a technique known as journey mapping to map the process of patient monitoring in each clinic and identify difficult or high-risk steps in the monitoring process [14]. Focusing on “pain points” identified through the journey mapping process for the Otolaryngology Clinic at the Zuckerberg San Francisco General Hospital, we applied a framework adapted from Systems Engineering Initiative for Patient Safety (SEIPS) [15] toward the development of a health IT tool that allows for context-specific customization of monitoring and treatment protocols for patients with head and neck cancer. This tool enables the clinic to develop custom diagnostic and treatment plans for patients with head and neck cancer, and facilitates subsequent population level tracking of completion of needed diagnostic or treatment steps.

We aim to implement the tool developed through this journey mapping process in the head and neck cancer clinic within a large safety net hospital and evaluate impact of this tool on timeliness of diagnostic evaluation, treatment initiation, and monitoring, as well as adherence to established protocols. Our objective here is to describe the study protocol for this pilot evaluation.

## Methods

### Study design

This study will evaluate implementation of a health IT tool designed to track patient progress toward diagnostic evaluation, treatment, and post-treatment monitoring for head and neck cancer. The study will evaluate the amount of time it takes patients to progress through key treatment milestones prior to and after implementation of the tool. The study design is a cohort study consisting of two cohorts of patients—a pre-treatment cohort including patients who have received a diagnosis of head and neck cancer but have not started treatment, and a post-treatment cohort of patients who have initiated treatment. Models controlling for secular trend will be used for data analysis to estimate the effect of tool implementation on improving timely and successful completion of guideline-based care processes. This analytic approach will help separate out the effect of the intervention from other temporal trends in care process completion. This study was approved by the University of California, San Francisco Institutional Review Board (12-09658) and is registered on ClinicalTrials.gov (Protocol ID: P30HS023558-1).

### Clinical setting

This study will take place within the Otolaryngology-Head and Neck Surgery Clinic at the Zuckerberg San Francisco General Hospital, a large county hospital affiliated with a tertiary care academic center. Patients served by this clinic are publicly insured. While the clinic has used an electronic health record for 22 years, it faces challenges, shared by many safety net systems, associated with coordinating care across multiple electronic platforms and record-keeping systems. Until adoption of this tool, the clinic did not have access to an integrated electronic registry system to monitor progress of care plans for patients undergoing diagnostic workup or treatment for head and neck cancers.

### Health IT tool

**Fig. 1:**
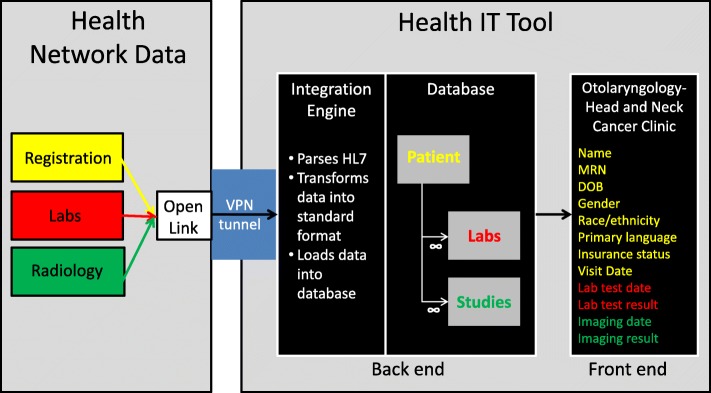
The health IT tool integrates data from three separate HL7 data sources representing registration, lab, and radiology data into a single database

The intervention evaluated in this study is an innovative workflow that incorporates an electronic registry tool. The tool integrates data from several, fragmented electronic medical records and allows clinic staff to monitor patient progress through care plans across the clinic’s entire panel (Fig. 1). Prior to development of this tool, the clinic relied on tracking methods requiring intensive manual data entry to populate a database that was not integrated with the medical record. These methods included a system of paper note cards and later an electronic spreadsheet to track patient progress. Using this tool requires changes in the clinic team’s workflow and communication.

**Fig. 2:**
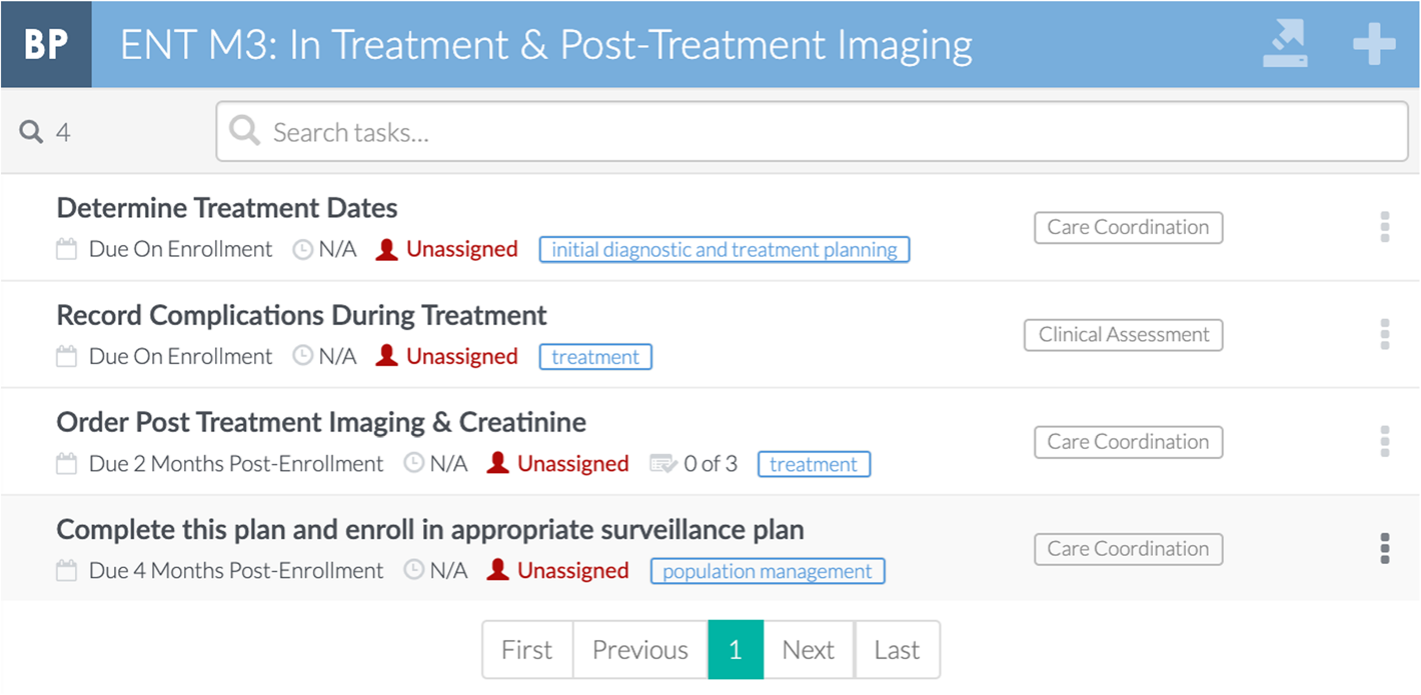
A screenshot depicting one of the clinic-specific treatment plans co-designed with the Otolaryngology-Head and Neck Surgery Clinic. After assigning a patient to this plan, care providers will be automatically prompted to complete its associated tasks, such as ordering post-treatment imaging for a patient two months after enrollment

**Fig. 3:**
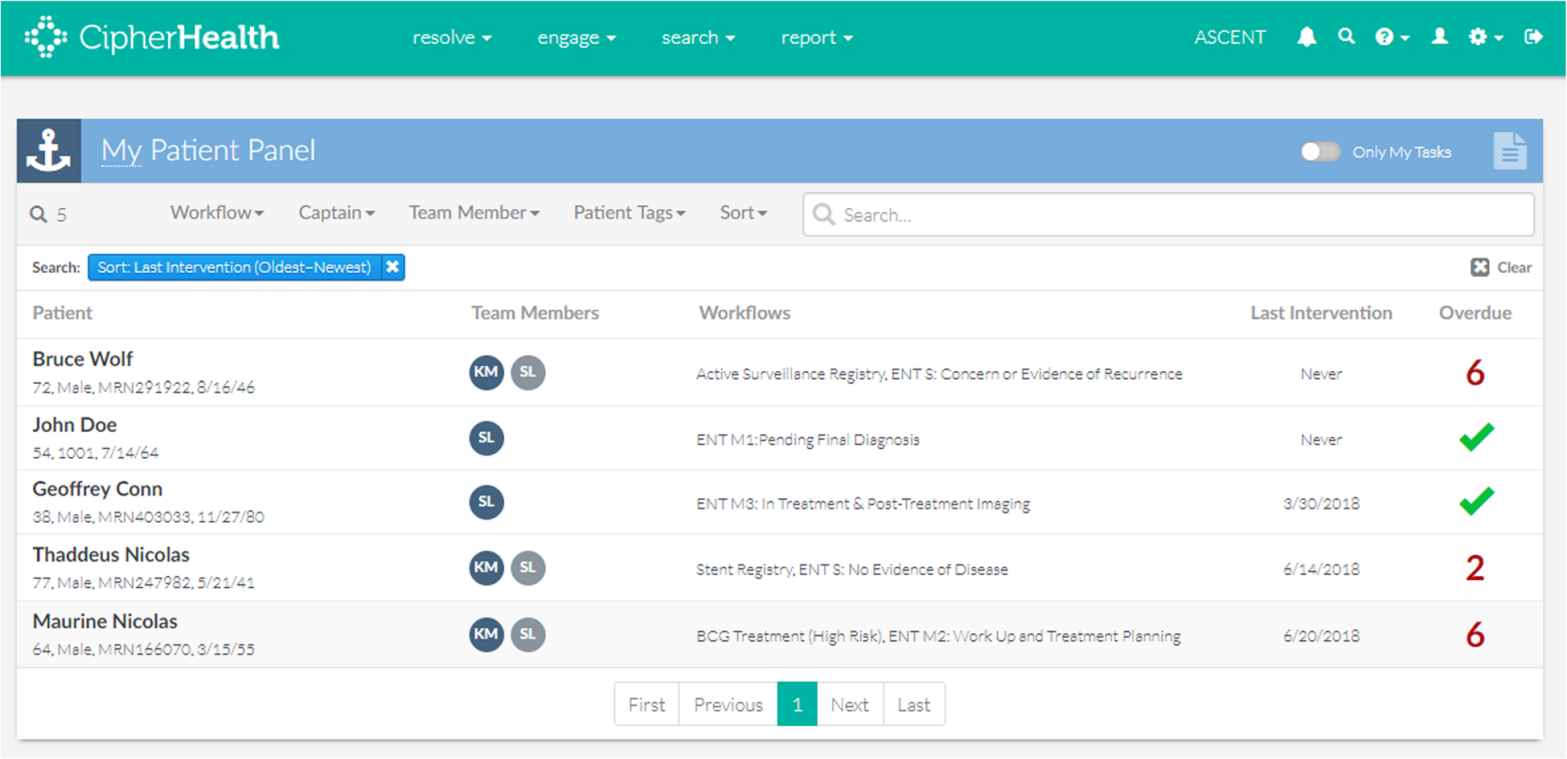
A screenshot of the health IT tool’s panel management functionality. Users can run queries to prioritize patients overdue for key steps of their assigned monitoring plan. Patient names are fictitious for demonstration purposes

The tool evaluated here was developed to address key process challenges identified through a “journey mapping” method with clinic staff in five clinics that monitor high-risk cancer situations: otolaryngology, pulmonary, breast, urology, and gastroenterology [14]. The journey mapping process identified two vulnerabilities present across all five sub-specialty clinics studied, namely (1) the need to track patient progress toward diagnostic or therapeutic goals and (2) the difficulty in creating comprehensive patient lists for monitoring. Practitioners in the Otolaryngology-Head and Neck Surgery Clinic also identified challenges related to coordinating multi-specialty treatment plans. Our team mapped processes identified during the journey mapping exercise onto the SEIPS model for work system, a model that describes the interrelated components of people, tasks, technology/tools, organization, and environment that make up clinical work systems [15]. We chose to focus on developing a solution to address challenges in work systems related to tasks and technology, as these were both the most clear areas of challenge and the most immediately amenable to change in the Otolaryngology-Head and Neck Surgery Clinic. In collaboration with CipherHealth, a health IT company, we developed a registry tool that integrates clinical data from multiple electronic health records. This tool allows for creation of clinic-specific treatment plans (Fig. 2) and tailored queries to identify patients who are overdue for completing key steps of their assigned treatment plan (Fig. 3).

### Chart review

Chart review will be conducted for all patients in the Otolaryngology-Head and Neck Surgery Clinic undergoing diagnostic workup, treatment, or post-treatment monitoring for head and neck cancer for the 2-year period leading up to implementation of the tool and the 2-year period after the tool has been implemented (with the 6 months prior to tool implementation excluded to avoid misclassification of exposure to the intervention, as discussed below). All patients with confirmed diagnosis of head and neck cancer who were seen in the clinic at least once will be included in the chart review. Based on prior clinic chart review, there are about 75 patients with head and neck cancer seen in the clinic each year who meet enrollment criteria. Thus, the chosen 4-year time period was selected to allow for inclusion of approximately 300 patients in our sample. Data will be extracted from patient charts and will include patient demographic factors, details of the diagnosis, and key treatment dates (Table 1). Unless noted, all clinical data will be collected from the medical record for both the intervention and control time periods, to minimize differential data collection methods that may influence comparisons.

**Table 1:** Data elements collected in chart review

Phase/data type	Data element
General: demographics	Gender
	Race/ethnicity
	Primary language
	Insurance status
General: social characteristics	Smoking status
	History of alcohol use
	History of other substance abuse
	History of homelessness/being marginally housed
	Comorbidities
	History of HIV/AIDS
General: utilization	Otolaryngology-Head and Neck Surgery visit dates and status (attended/canceled/no-show/scheduled)
	Medical oncology visit dates and status (attended/canceled/no-show/scheduled)
	Radiation oncology visit dates and status (attended/canceled/no-show/scheduled)
	Patient only seen for hospitalization
	Most recent visit date
General: outcomes	Overall follow-up time
	Survival
General: results	Imaging for cancer monitoring
	All imaging
	Thyroid-stimulating hormone test dates and results
	All lab test dates and results
	Pathology dates and results
1. Workup and treatment planning	Date of diagnosis
	Date of referral to tumor board
	Date of presentation at tumor board
	Date imaging appointments sent, scheduled, and completed
	Date referrals sent, scheduled, and completed
	TNM staging
	Site
	Histology
	P16 test results*
	Treatment plan
	Dates of patient outreach^†^
	Date of recurrence
2. In treatment	Date of dental evaluation
	Date of treatment start
	Date of treatment completion
	Recommended and received dose and treatment sessions
	Treatment result (e.g., completed, not completed, delays)
	Dates of treatment delays/complications
	Type of treatment delays/complications
	Dates post-treatment imaging ordered and completed
	Dates of patient outreach^†^

*P16 immunohistochemistry tests are recommended for newly diagnosed oropharyngeal squamous cell carcinomas

^†^Supplemental data collected from health IT tool to describe implementation of the tool, all other data collected from chart review

### Outcomes

Patients included in the study will be divided into two separate cohorts. The pre-treatment cohort will include patients with a confirmed diagnosis of head and neck cancer but who have not yet initiated treatment. The post-treatment cohort will include patients who have initiated treatment and are undergoing additional treatment modalities and monitoring. Patients who received a diagnosis of head and neck cancer and initiated any treatment will thus be present in both cohorts. Outcomes for each cohort consist of completion of key steps in the evaluation and treatment process, and are summarized in Table 2. Usual clinic protocols will be employed to address patients who are lost to follow-up (three outreach attempts, then moved to lost to follow-up list). Patients who are lost to follow-up will be included in the final analysis and will be considered to have not met the process outcome for their given cohort (i.e., it will be assumed that patients lost to follow-up from the pre-treatment cohort never initiated treatment).

**Table 2:** Primary and secondary outcomes for each patient cohort

Cohort	Primary outcome	Secondary outcomes
Pre-treatment cohort: workup and treatment planning	- Time from diagnosis to initiation of treatment	- Time from diagnosis to presentation to tumor board - Time from diagnosis to dental evaluation - Time to first visit in medical oncology and radiation oncology clinics - Lost to follow-up (no visit for ≥ 6 months)
Post-treatment cohort: treatment	- Completion of tumor board treatment recommendations	- Lost to follow-up (no visit for ≥ 6 months) - Proportion of patients completing post-treatment radiation within 6 weeks (guideline-based treatment quality of care metric) [5, 6]

### Classification of exposure to the intervention

Patients will be considered unexposed to the intervention if they entered one of the cohorts at least six months prior to implementation of the health IT tool intervention. Patients who entered a cohort less than 6 months prior to implementation of the intervention will be excluded from that cohort. All patients entering a cohort after implementation of the tool will be considered exposed to the intervention for that cohort. For example, if a patient was diagnosed with head and neck cancer 3 months prior to implementation of the intervention and initiated treatment 1 week after implementation of the intervention, they would be excluded from the pre-treatment cohort and would be included in the post-treatment cohort and considered exposed to the intervention.

### Implementation outcomes

The key implementation outcome is feasibility. We will also measure several components of tool utilization to better understand the actor, dose, temporality, and action target of the intervention [16]. Data collection on these parameters will occur through a quarterly survey of clinic staff using the tool and through five randomly selected clinic days when the investigators will observe clinic staff and any use of the tool that occurs. The survey will ask staff to report their role in the clinic and recall for the prior week the amount of time that the tool was used, timing of use, and number of patients outreached through the use of the tool. The feasibility objective for this pilot study is to achieve consistent use throughout all clinic sessions reaching all eligible patients. The feasibility outcome which will determine success and trigger proceeding to the main trial is achievement of significant use: use during at least 80% of clinic sessions and for 80% of eligible patients.

### Analysis

Incorporation of a term for calendar time into our models for primary and secondary outcomes will allow us to control for secular trend. This strengthens the analysis by eliminating temporal improvements in clinical care processes that are unrelated to the intervention. Time to event analyses will use Cox proportional hazards models. For binary outcomes including loss to follow-up and completion of treatment steps such as post-treatment radiation within 6 weeks, logistic regression will be used. Intervention effect estimates will be adjusted for potential confounders including patient demographics, substance use, housing status, and cancer stage.

### Minimum detectable effects

In Cox models for time to event, the sample of 300 will provide 80% power within two-sided tests with alpha of 0.05 to detect a hazard ratio of 2.25 for the effect of the intervention, after adjusting for a linear temporal trend as well as confounders. Estimation of preliminary effects should be considered secondary objectives. The sample size we propose is based on feasibility outcomes, and therefore the simulations below demonstrate that the pilot sample size would only detect a substantial effect size. Depending on the feasibility outcomes, we would proceed to a larger trials with the ability to detect more modest effects. With the present number of patients, in Cox models for time to event, the sample of 300 will provide 80% power within two-sided tests with alpha of 0.05 to detect a hazard ratio of 2.25 for the effect of the intervention, after adjusting for a linear temporal trend as well as confounders. For binary outcomes including loss to follow-up and completion of stages, it will provide 80% power to detect intervention odds ratios of 3.4 to 6.7, depending on the number of patients included in the analysis (200–300) and the prevalence of the outcome (20 to 50%), again after adjusting for a linear temporal trend and confounders. These estimates were obtained using simulations implemented in R Version 3.4.3.

## Discussion

Health IT and management tools can improve the quality and safety of care delivered [17]. The tool designed in this study has the potential to improve patient safety in diagnostic, treatment, and monitoring steps for management of patients with head and neck cancer. By mapping out care processes in conjunction with frontline clinicians and incorporating this information with the systems engineering perspective provided by the SEIPS framework, we developed a tool that clearly addresses challenging aspects of providing care that both “keep clinicians up at night” and contribute to poor clinical outcomes. This study will evaluate the effectiveness of this tool for improving timely delivery of care, and results will inform the development of a multi-center trial to evaluate effectiveness of this tool in a broader array of healthcare settings.

Although our study is limited somewhat by the retrospective nature of our pre-intervention comparison, we plan to address this limitation by adjusting for confounding by other temporal trends during the period of comparison. The study is also unable to evaluate patient progress towards diagnosis once referred to the Otolaryngology-Head and Neck Surgery Clinic. Though the tool will be used to monitor this aspect of care, there is no reliable way to identify the pre-intervention comparison cohort.

Ultimately, we hope that this tool will be adapted to other clinical contexts to expand the ability of clinics and care providers to track and intervene on patients not meeting treatment and monitoring goals. We plan to publish study results in the scientific literature, as well as compile and share results with the Otolaryngology-Head and Neck Surgery Clinic included in this study.

## Data Availability

The datasets used and/or analyzed during the current study are available from the corresponding author on reasonable request.

## Abbreviations

IT: Information technology
NCCN: National Comprehensive Cancer Network
SEIPS: Systems Engineering Initiative for Patient Safety
